# Extirpation of the Parotid Gland

**Published:** 1832

**Authors:** 


### II. ANALYTICAL.

26. *Extirpation of the Parotid Gland.*—This formidable operation has been lately performed with partial success by Dr. Mott, of New York. S. B. of St. Domingo, applied to Dr. Mott in the latter part of June, of last year, for advice respecting a tumor about the size of an ordinary fist, involving nearly the whole of the left side of the face. The swelling had attracted the attention of the patient about six months before, and was apparently of a schirrous character, originating in the parotid gland.

The operation was performed on the 13th of July. The external carotid which, in consequence of the swelling of the integuments, lay three inches below the surface was tied just below the digastric muscle. An incision was next made from a point above the jugum, passing downwards in a semicircular direction to the occipital bone, and the incision above the artery carried upward to intersect this.

When the integuments were turned backward, the dark appearance of the gland shewed that the tumor, instead of being shcirrous, was melanotic. The cellular tissue was divided until the masseter muscle was exposed to view, from which it was separated for some distance; it was then separated from the jugum which had become carious from pressure; having detached it below from the mastoid and digastric muscles, an attempt was made to elevate the tumor, but this attempt produced such excruciating pain that the dissection was completed by cutting from above downwards. The portion between the mastoid and styloid processes was cautiously separated with the handle of the scalpel, and the portio dura divided by a quick motion of the knife. The division of this nerve produced very severe pain, and paralysed the muscles of the face.

Several arteries were tied, and, among others, the temporal poured forth a profuse retrograde hæmorrhage.

The wound healed kindly, except a small point which, on the 27th, began to give evidence of a return of the disease. On the 5th August, the re-united portions of the wound began to throw out a melanoid fungus, and tumors of the same character appeared on different parts of the face and scalp. The symptoms increased, an hepatic affection supervened, and he died on the 5th of September.

The practicability of this operation may now be considered as fully established. The success of the operator, in this case, so far as the removal of the gland is concerned, was complete, and the death of the patient is evidently to be attributed to constitutional disease.—*Am. Jour. of Med. Sciences.*

